# Prognostic value of tertiary lymphoid structures in triple-negative breast cancer: integrated analysis with the tumor microenvironment and clinicopathological features

**DOI:** 10.3389/fimmu.2024.1507371

**Published:** 2024-12-12

**Authors:** Florence Boissière-Michot, Marie-Christine Chateau, Simon Thézenas, Virginie Lafont, Evelyne Crapez, Priyanka Sharma, Angélique Bobrie, Pascal Roger, Séverine Guiu, William Jacot

**Affiliations:** ^1^ Translational Research Unit, Montpellier Cancer Institute Val d’Aurelle, Montpellier, France; ^2^ Biometry Department, Montpellier Cancer Institute Val d’Aurelle, Montpellier, France; ^3^ Institut de Recherche en Cancérologie de Montpellier (IRCM), Inserm U1194, Montpellier, France; ^4^ Department of Medical Oncology, Montpellier Cancer Institute Val d’Aurelle, Montpellier, France; ^5^ Pathology Department, Nîmes University Hospital, Nîmes, France; ^6^ Faculty of Medicine, Montpellier University, Montpellier, France

**Keywords:** tertiary lymphoid structures, triple-negative breast cancer, tumor immune microenvironment, prognostic biomarker, predictive biomarker

## Abstract

**Background:**

In triple-negative breast cancer (TNBC), the most immunogenic breast cancer type, tumor-infiltrating lymphocytes (TILs) are an independent prognostic factor. Tertiary lymphoid structures (TLS) are an important TILs source, but they are not integrated in the current prognostic criteria.

**Methods:**

In this retrospective study, TLS were assessed in hematein-eosin-saffron-stained (HES) histological sections from 397 early, chemotherapy-naive TNBC samples after primary surgical resection. Their association with i) classical clinicopathological features, ii) TILs and CD3+, CD8+, CD20+ lymphoid populations, iii) CD68+, CD163+, CD11b+, CD66b+ myeloid populations, and iv) expression of the PD1/PD-L1 and PVR/TIGIT axis immune checkpoint components and their prognostic significance were evaluated.

**Results:**

TLS were observed in 88.2% of samples, mainly in peritumoral areas (86.1%). Increased amount of peritumoral TLS (PT-TLS) was significantly associated with younger age (p<0.001), smaller tumor size and higher tumor grade (both, p<0.001), HER2^null^ tumors (versus HER2^low^ tumors, p<0.002), and non-lobular histological type (p<0.016). TNBC with higher PT-TLS abundance displayed more often a basal-like (p<0.001) and not molecular-apocrine phenotype (p<0.001). TLS abundance was associated with TILs and hot tumor inflammatory pattern (both, p<0.001). Remarkably, PT-TLS abundance was positively associated with the density of the analyzed lymphoid (CD3+, CD8+, CD20+) and myeloid (CD68+, CD163+, CD11b+) cell populations (all p<0.001), with the exception of CD66b+ cells, as well as with expression of the PD1/PD-L1 and TIGIT/PVR immune checkpoint markers. In univariate analysis, beside the classical clinicopathological factors (tumor size, node involvement and adjuvant chemotherapy), TILs, hot tumors and PT-TLS were significantly associated with clinical outcome. Moreover, the risk of relapse was inversely correlated with PT-TLS abundance (Kaplan-Meier analysis). In multivariate analysis, pathological stage, adjuvant chemotherapy and PT-TLS remained correlated with relapse-free survival.

**Conclusion:**

Our results suggest that TLS are a frequent feature in early TNBC and that their presence, particularly at the tumor periphery, recapitulates the tumor immune microenvironment. In our series, their prognostic value outperformed that of TILs. Therefore, their easy quantification on routine HES sections and their integration into the factors classically analyzed by pathologists could improve the clinical management of TNBC, a breast cancer type whose prognosis remains too poor.

## 1 Introduction

Triple-negative breast cancer (TNBC) accounts for ~15% of breast cancers and is the most aggressive subtype. Although all TNBCs are characterized by the absence of estrogen (ER) and progesterone receptors (PR) expression and lack of HER2 overexpression, they display strong inter- and intra-tumoral heterogeneity, as highlighted by genomic (1, 2) and phenotypic analyses (3, 4). Until recently, their treatment relied on the triad of surgery, chemotherapy and radiotherapy, but advances in targeted therapies have transformed the therapeutic landscape. Since 2021, immunotherapy associated with chemotherapy has become the standard of care for patients with localized stage (≥T2 and/or N+) and metastatic TNBC (5). Moreover, sacituzumab govitecan, an antibody-drug conjugate that targets human trophoblast cell-surface antigen 2 coupled to the topoisomerase I inhibitor SN-38, has been approved for patients with metastatic TNBC after two or more lines of systemic therapy (6). Despite these advances, the recurrence rate remains high and the prognosis poor.

While the efficacy of immunotherapy is limited to a subset of patients, there are currently no robust biomarkers to rationalize treatment choices, particularly in the case of localized breast cancer (7). For example, the anti-PD-1 antibody pembrolizumab has been approved in combination with chemotherapy for the neoadjuvant treatment of patients with TNBC at high risk of recurrence (≥T2 or N+ stage), whatever the PD-L1 status (8). Conversely, the same combination has been approved for the treatment of patients with unresectable or metastatic TNBC only if the PD-L1 combined positive score is ≥ 10 (9). Therefore, additional biomarkers of prognosis and ideally, predictive of the therapeutic efficacy (especially of immunotherapy that has delicate side effects profile) are needed.

Recently, the presence of mature tertiary lymphoid structures (m-TLS) has been associated with the response to immunotherapy in various solid cancers (10, 11). TLS are ectopic lymphoid aggregates of B and T cells admixed with dendritic cells (DCs) that are formed in inflammatory contexts, such as chronic inflammation (12), autoimmune diseases (12) and cancer (13). TLS are associated with worse prognosis in chronic inflammatory and autoimmune diseases. Conversely, their presence is usually associated with favorable clinical outcome in cancer. TLS have been observed in various solid tumors, including TNBC (14–17), but data are scarce and not always consistent, probably because there was no clear definition of how a TLS should be identified. As TLS presence is emerging as a promising biomarker, efforts have been made to standardize their identification in cancer in a way that can be used in the clinic (18).

TLS contribute to the cancer immune surveillance by sustaining B-cell activation, maturation and selection, by producing tumor-relevant antibodies, and by presenting tumor antigens to T cells (10). They represent the principal source of Tumor-Infiltrating Lymphocytes (TILs). Interestingly, TNBCs are highly immunogenic tumors characterized by a high mutation burden and the strongest immune infiltrate among all breast cancer types. It is broadly acknowledged that in TNBC, high TIL abundance is associated with improved survival (19, 20). Therefore, TILs are now routinely assessed (21, 22), but not TLS despite their undisputed central role in TNBC biology.

Here, using samples from a well-characterized cohort of 397 patients with localized chemotherapy-naive TNBC, we retrospectively evaluated TLS localization/abundance and their relationship with clinical-pathological features and clinical outcome. Our objective was i.) to better describe the interactions between TLS and the different cell populations of the tumor microenvironment, ii.) to better understand the relationship between TLS presence and the phenotypic TNBC subgroups, and iii) to assess their impact on the clinical outcome.

## 2 Materials and methods

### 2.1 Study design

This study received full ethical approval by the appropriate regulatory bodies, particularly the Institutional Review Board (approval N° ICM-CORT-2022-11). The study followed the ethical principles of the Declaration of Helsinki. All patients included in the study were informed that their biological samples could be used for research purposes, and were given the opportunity to object. All the samples belonged to a collection at our institution’s Biological Resources Center, declared (DC-2024-6524) and authorized (AC-2019-3607) by the French Ministry of Research, which certifies that the use of the samples complies with current legislation (Article R1243-49 of the French Public Health Code).

Most TNBC samples (92%) used in this study were from a single-center series (Institut du Cancer de Montpellier, France) and the others (8%) were from the pathology department of Nîmes University Hospital, France. Included patients had unifocal, unilateral, localized TNBC without neo-adjuvant chemotherapy and without history of another invasive cancer in the previous 5 years. TNBC were defined as tumors with <10% of cancer cells that expressed ER and PR by immunohistochemistry (IHC), HER2 0, 1+ or 2+ by IHC, and for HER2 2+ tumors, absence of *ERBB2* gene amplification by *in situ* hybridization. Each patient was treated in accordance with our institution’s guidelines. Patients’ data (age, tumor size, nodal status, histological grade and type, HER2 status, adjuvant chemotherapy and clinical follow-up) were collected retrospectively by chart review and were summarized by descriptive analysis (**Table 1**). Samples from 2002 to 2010 fulfilling the inclusion/exclusion criteria were selected from a prospective institutional breast cancer database (tumor biobank number BB-0033-00059). Samples from 2011 to 2018, meeting the same inclusion criteria, were identified retrospectively from pathology department data and the analysis of clinical records. In all, 467 TNBC samples were selected, de-identified and arrayed on nine tissue-microarrays (TMA; 2 cores of 1mm in diameter per sample). Finally, 397 TNBC samples from patients who met all inclusion criteria and for whom an assessable Hematein-Eosin-Saffron (HES)-stained section of the whole tumor was available were included.

**Table 1:** Clinicopathological features of the patients with TNBC.

	N = 397	%
Age (years), median [min-max]	59.2 [27.0-98.7]	
Tumor size		
T1	191	48.0
T2	184	46.2
T3/T4	23	5.8
Node status		
N-	269	67.8
N+	128	32.2
Pathological stage		
I	153	38.5
II	187	47.1
III	57	14.4
Histological grade		
I / II	69	17.6
III	324	82.4
HER2		
Null	313	78.8
Low	84	21.2
Histology		
No special type	340	85.7
Lobular	16	4.0
Other	41	10.3
Adjuvant chemotherapy (missing: 2)		
No	94	23.8
Yes	301	76.2
Basal-like phenotype (missing: 7)		
Basal-like	234	60.0
Non-basal-like	156	40.0
Molecular apocrine phenotype (missing: 28)		
Molecular apocrine	133	36.0
Non-molecular apocrine	236	64.0
TILs		
<30%	261	65.7
≥30%	136	34.3
Inflammatory pattern		
Cold	232	58.4
Excluded	59	14.9
Hot	106	26.7
Peri- and/or intratumoral TLS		
Present	350	88.2
Absent	47	11.8
Peritumoral TLS		
None	55	13.9
Little	87	21.9
Moderate	178	44.8
Abundant	77	19.4
Intratumoral TLS		
Absent	299	75.3
Present	98	24.7
m-TLS (with CGC)		
Absent	255	72.9
Present	95	27.1

Basal-like phenotype was defined by the expression of cytokeratin 5/6 and/or EGFR in >10% of tumor cells; molecular apocrine phenotype was considered in the case of positive staining for both androgen receptor and Forkhead box protein A1 (FoxA1) biomarkers in ≥1% of tumor cells. TILs, tumor-inflitrating lymphocytes; TLS, tertiary lymphoid structures; m-TLS, mature-TLS; CGC, clear germinal center.

### 2.2 Histopathological evaluation

The most representative tumor block of each surgical specimen was selected to assess TLS and TILs. HES-stained full-face sections were evaluated by a board-certified pathologist (M-CC) blinded to all other clinicopathological data.

According to Vanhersecke et al. (18), TLS were defined as lymphoid aggregates of more than 50 immune cells localized in the peritumoral (PT) or intratumoral area (IT). Each tumor was classified using the tumor circumference occupied by TLS, within 1 mm of the tumor front, as proposed by Lee et al. (14): none (no TLS identified in the PT area), little, moderate and abundant (1-10%, 11-50% and >50% of the tumor circumference occupied by TLS, respectively). The presence of intratumor TLS (IT-TLS) outside necrotic and ulcerated areas was also reported as well as the presence or absence of clear germinal centers (CGC) that indicates the mature nature of TLS.

Tumors were also classified according to their inflammatory pattern: “cold” when the tumor parenchyma and stroma were devoid of lymphocytic infiltration; “hot” when the stromal and epithelial compartments were infiltrated by lymphocytes; or “excluded” when inflammation was limited to the tumor stroma or periphery (23).

TILs were quantified using semi-continuous 10% increments as recommended by the International Immuno-Oncology Biomarker Working Group guidelines (22).

### 2.3 Immunohistochemistry

**Figure 1:**
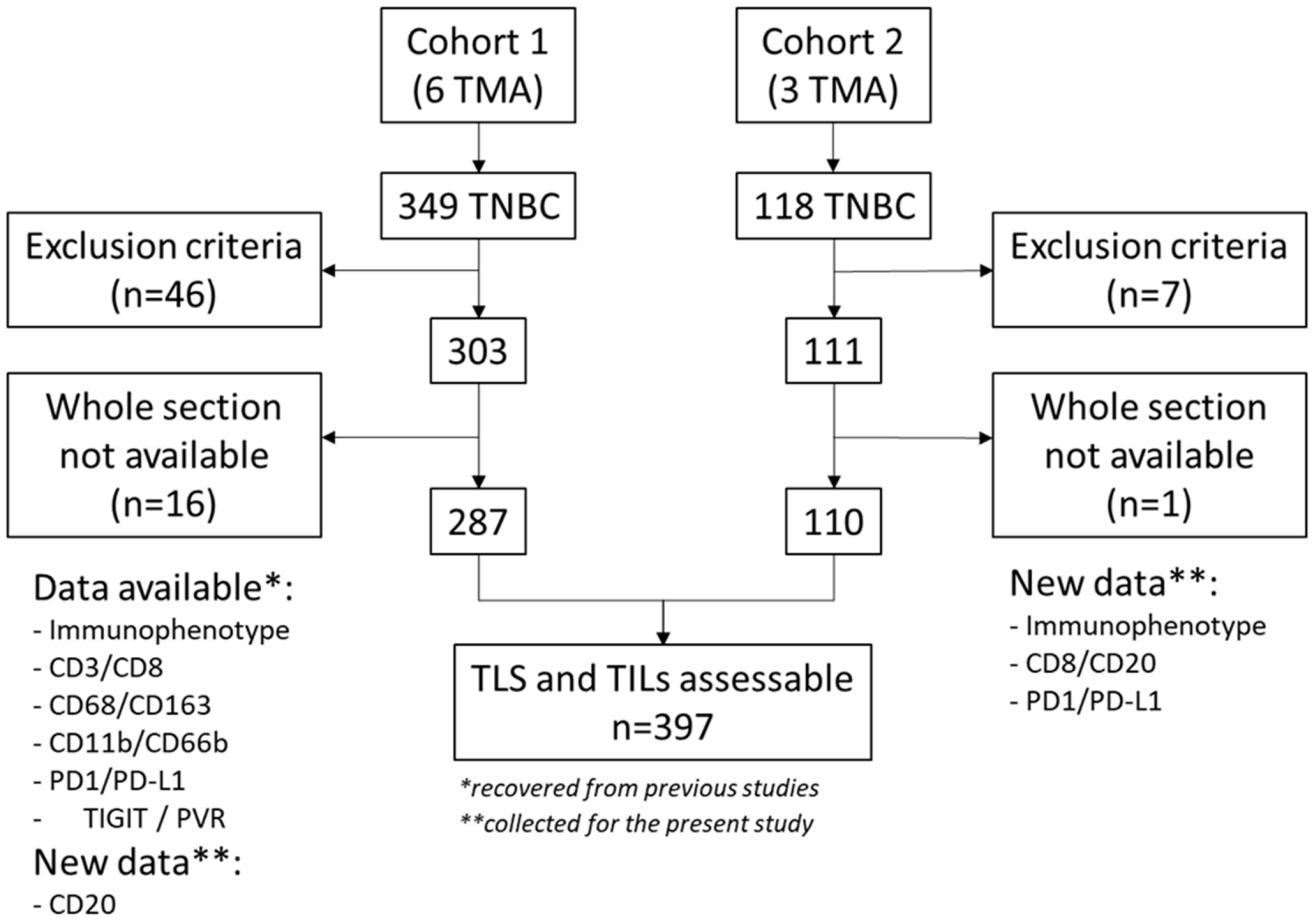
Consort flow diagram. TLS and TILs assessable n=397, the asterisk * refers to biomarkers recovered from previous studies while the asterisk ** refers to data collected for the present study.

Data on most of the biomarkers used in this study were recovered from previously published studies by our group on 349 patients (TNBC samples in six TMAs) (**Figure 1**). TLS could be analyzed on 287 samples. The detailed procedures are described in these previous studies (3, 24–26). The cohort was completed by 118 other TNBC specimens arranged on three new TMAs where TLS could be analyzed in 110 samples (**Figure 1**). To obtain data on CD8+ cells, PD1 and PD-L1 expression and on androgen receptor (AR), FOXA1, CK5/6 and EGFR (required for sub-group phenotyping), the three new TMAs were analyzed using the same IHC and quantification procedures as previously described (3, 24). Given the crucial role of TLS in B-cell recruitment and differentiation, the B-cell infiltrate was assessed by IHC using the mouse monoclonal ready-to-use anti-CD20 antibody (clone L26, Roche) and a Ventana Discovery IHC device, according to the supplier’s recommendations. The density of CD20+ cells, expressed as the number of CD20+ cells/mm², was quantified on digitized slides (Nanozoomer scanner, Hamamatsu) in TMA cores with the Histolab^®^ software, as previously described (27). Some samples could not be analyzed because of core loss during processing, poor quality, or presence of only benign tissue in the core.

For statistical analysis and for each immune population, tumors were divided in ‘‘low’’ and ‘‘high’’ density using the median as cut-off value, except for PVR for which the first two terciles defined the ‘‘low’’ density and the third tercile defined the ‘‘high’’ density, according to previously published data (25).

### 2.4 Statistical analysis

Categorical variables were reported by means of contingency tables. For continuous variables, median and range were computed. To investigate their associations with clinical, pathologic and biologic parameters, univariate statistical analyses were performed using the Pearson’s chi-square or Fisher’s exact test when applicable for categoric variables, and the Kruskal-Wallis or Student’s *t* test for continuous variables. Survival data were estimated with the Kaplan-Meier method and were presented as median and rate, with 95% confidence intervals (CI). The median follow-up was estimated using the reverse Kaplan-Meier method and presented with its 95% CI. Relapse-free survival (RFS) was defined as the time from the date of surgery to the date of the first documented tumor relapse (local and/or distant). In this population with long follow-up, a significant number of deaths was unrelated to the cancer. Therefore, for this study, RFS was considered a better clinical end-point than overall survival. Patients alive without an event were censored at the last known date of a follow-up visit. Survival curves were drawn and the log-rank test was performed to assess differences between groups. Multivariate analyses were carried out using Cox proportional hazards regression models, with a stepwise selection procedure, to investigate known prognostics factors. Hazard ratios (HR) with their 95% CI are presented to display the risk reduction. All P values were two sided, and the significance level was set at 5% (p<0.05). Statistical analyses were performed with the STATA 16.1 software (Stata Corporation, College Station, TX).

## 3 Results

### 3.1 Patient and tumor characteristics

**Table 1** describes the main clinical-pathological characteristics of the 397 patients with TNBC samples in which TLS could be analyzed. Their median age was 59.2 years [27.0 to 98.7 years]. In most patients, TNBC was high-grade (82.4%), of no special type (85.7%), mostly without lymph node involvement (67.8%). Moreover, 21.2% of samples displayed HER2^low^ status (1+ by IHC or 2+ by IHC without *ERBB2* gene amplification). According to our institution guidelines, 76.2% of patients received adjuvant chemotherapy. In addition, 60% of TNBC samples were classified as basal-like (based on CK5/6 and/or EGFR expression in >10% of tumor cells) and 36% had a molecular apocrine phenotype (based on positive staining for AR and FOXA1 in ≥1% of tumor cells).

According to Salgado’s criteria (22), 34.3% of samples had stromal infiltration with ≥30% of TILs. Moreover, 58.4%, 14.9%, and 26.7% of TNBC samples were categorized as cold, immune-excluded and hot, respectively.

### 3.2 TLS characterization

**Figure 2:**
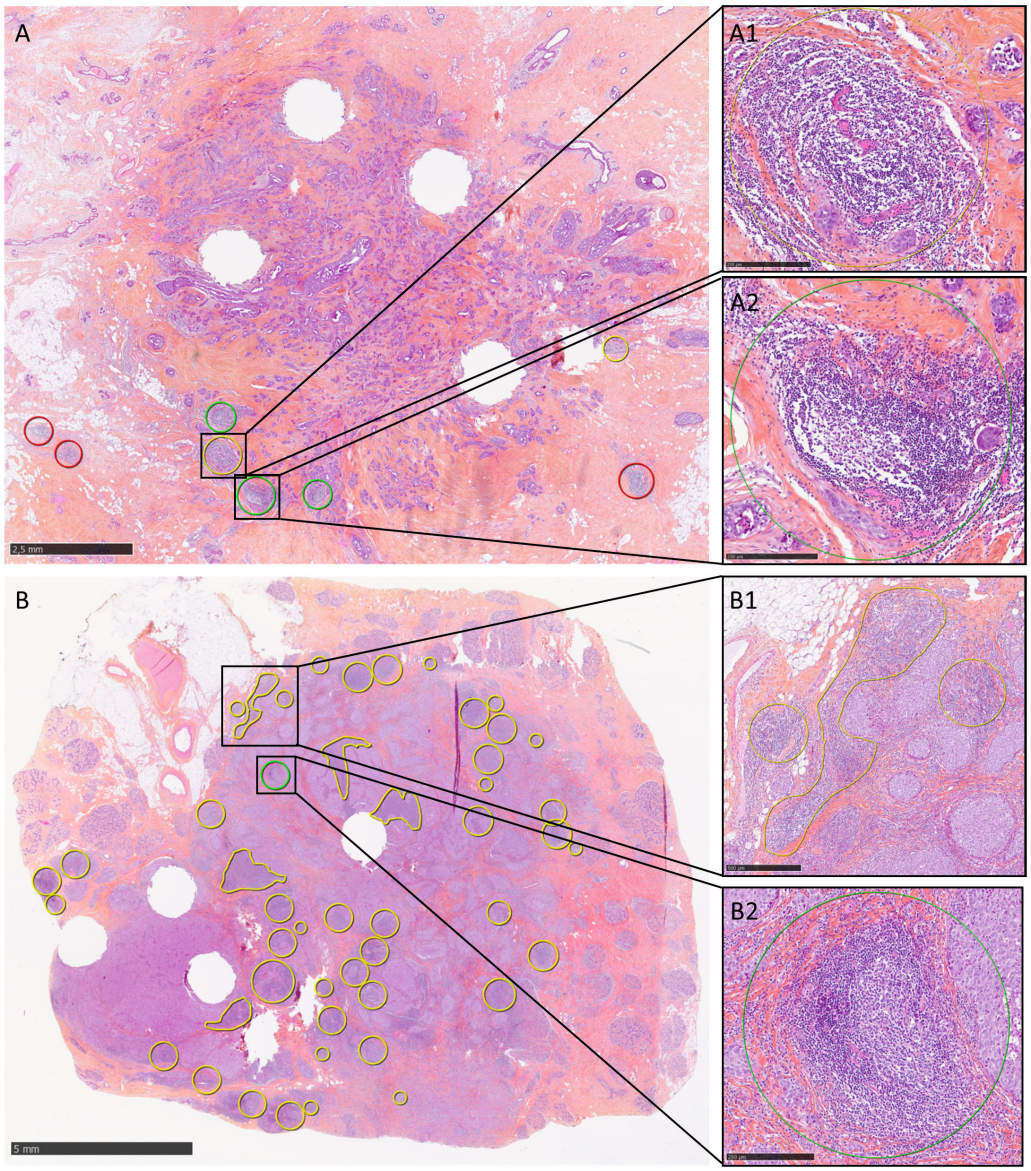
Representative images of TLS detection in two TNBC samples. **(A)** TLS are located exclusively at the tumor periphery (TLS circled in red were not evaluated because located at >1 mm from the tumor front) and occupy <10% of the tumor circumference. Some TLS show no CGC (yellow circle, A1), while others do (green circle, A2). **(B)** TLS are present both in the tumor parenchyma and at the periphery, where they occupy between 10% and 50% of the tumor circumference. TLS may be nodular or sheet-like (B1). In this sample, a CGC was observed in only one intratumoral TLS (B2). Scale bars: A, 2.5 mm; B, 5 mm; A1, A2, B2, 250 µm; B1, 500 µm.

TLS were observed in 88.2% (350/397) of the studied TNBC samples (**Table 1**; **Figure 2**). TLS were mainly at the periphery of the invasive component (86.1%; 342/397 samples): 21.9%, 44.8% and 19.4% of samples showed little, moderate and abundant PT-TLS, respectively. TLS were less frequently observed within the tumor parenchyma (24.7%; 98/397 samples). Only 8 samples displayed TLS exclusively within the tumor parenchyma (IT-TLS). In the 350 samples with at least one TLS (PT- or IT-TLS), TLS with CGC were detected in 95 tumors (27.1%) and were considered as m-TLS.

### 3.3 Association between TLS and clinicopathological data (univariate analysis)

The results of the univariate analysis of TLS abundance/features and clinicopathological data are presented in **Table 2**. PT-TLS abundance decreased with age (p<0.001, **Table 2**; **Supplementary Figure S1A**). Conversely, IT-TLS and CGC presence/absence were similar in younger and older patients (**Table 2**; **Supplementary Figures S1B, C**). PT-TLS abundance was higher in small tumors and high-grade tumors (both, p<0.001) and high pathological stage tended to be associated with less PT-TLS (p=0.059). PT-TLS abundance was lower (mainly none and little) in lobular TNBC than in the other types (p=0.016). High levels of PT-TLS were more frequently observed in HER2^null^ than HER2^low^ tumors (88.3% of samples with abundant PT-TLS were HER2^null^). Similarly, 91.6% of samples with m-TLS (identified by the presence of CGC) were HER2^null^. The basal-like phenotype was more frequent in samples with higher PT-TLS abundance (p<0.001). Conversely, the molecular apocrine phenotype was more often observed in samples with little or no PT-TLS (p<0.001), and in tumors with IT-TLS (p=0.024). This means that moderate and abundant PT-TLS were more frequent in basal-like and non-molecular apocrine tumors than in non-basal-like or molecular apocrine tumors (**Supplementary Figure S2**). PT-TLS (p<0.001) and m-TLS (p=0.007) were more frequently detected in TNBC samples of patients who received adjuvant chemotherapy compared with those who did not. This may reflect the association between adjuvant chemotherapy prescription, younger age and higher tumor grade that are also associated with PT-TLS presence. Lastly, no statistically difference in TLS abundance (PT-TLS, IT-TLS, with or without CGC) was observed between N+ and N- TNBC samples, although more tumors without PT-TLS were N+ (43.6% of tumors without PT-TLS were N+ versus 29.9%, 32.0% and 27.3% with little, moderate, abundant PT-TLS, respectively).

**Table 2:** Results of the univariate analysis of association between TLS features and clinicopathological data.

	Peritumoral TLS					Intratumoral TLS			TLS		
	None	Little	Moderate	Abundant	p-value	No	Yes	p-value	Without CGC	With CGC	p-value
	n=55	n=87	n=178	n=77		n =299	n =98		n =255	n =95	
Age (years)					<0.001			0.977			0.158
<59.2	19 (34.5%)	41 (47.1%)	85 (47.8%)	54 (70.1%)		150 (50.2%)	49 (50.0%)		126 (49.4%)	55 (57.9%)	
≥59.2	36 (65.5%)	46 (52.9%)	93 (52.2%)	23 (29.9%)		149 (49.8%)	49 (50.0%)		129 (50.6%)	40 (42.1%)	
Mean ± SD	69.9 ± 13.2	61.1 ± 14.4	59.1 ± 15.1	54.2 ± 12.9	<0.001	60.0 ± 14.5	57.6 ± 14.6	0.166	59.7 ± 15.1	56.5 ± 13.3	0.088
Tumor size					<0.001			0.384			0.955
T1	21 (38.2%)	35 (40.2%)	88 (49.4%)	47 (61.0%)		144 (48.2%)	47 (47.9%)		125 (49.0%)	48 (50.5%)	
T2	24 (43.6%)	47 (54.0%)	84 (47.2%)	28 (36.4%)		135 (45.1%)	48 (49.0%)		120 (47.1%)	43 (45.3%)	
T3/T4	10 (18.2%)	5 (5.8%)	6 (3.4%)	2 (2.6%)		20 (6.7%)	3 (3.1%)		10 (3.9%)	4 (4.2%)	
Node status					0.225			0.550			0.991
N-	31 (56.4%)	61 (70.1%)	121 (68.0%)	56 (72.7%)		205 (68.6%)	64 (65.3%)		177 (69.4%)	66 (69.5%)	
N+	24 (43.6%)	26 (29.9%)	57 (32.0%)	21 (27.3%)		94 (31.4%)	34 (34.7%)		78 (30.6%)	29 (30.5%)	
Pathological stage					0.059			0.414			0.961
**I**	19 (34.6%)	30 (34.5%)	68 (38.2%)	36 (46.7%)		116 (38.8%)	37 (37.7%)		98 (38.4%)	38 (40.0%)	
**II**	23 (41.8%)	39 (44.8%)	90 (50.6%)	35 (45.5%)		144 (48.2%)	43 (43.9%)		123 (48.3%)	45 (47.4%)	
**III**	13 (23.6%)	18 (20.7%)	20 (11.2%)	6 (7.8%)		39 (13.0%)	18 (18.4%)		34 (13.3%)	12 (12.6%)	
Histological grade	*(missing: 4)*				<0.001	*(missing: 4)*		0.066			0.083
I / II	20 (36.4%)	21 (24.4%)	22 (12.5%)	6 (7.9%)	<0.001	46 (15.5%)	23 (23.7%)		43 (17.1%)	9 (9.6%)	
III	35 (63.6%)	65 (75.6%)	154 (87.5%)	70 (92.1%)		250 (84.5%)	74 (76.3%)		209 (82.9%)	85 (90.4%)	
HER2					0.002			0.621			<0.001 <0.001
Null	40 (72.7%)	58 (66.7%)	147 (82.6%)	68 (88.3%)		234 (78.3%)	79 (80.6%)		193 (75.7%)	87 (91.6%)	
Low	15 (27.3%)	29 (33.3%)	31 (17.4%)	9 (11.7%)		65 (21.7%)	19 (19.4%)		62 (24.3%)	8 (8.4%)	
Histology					0.016			0.131			0.107
No special type	43 (78.2%)	69 (79.3%)	161 (90.5%)	67 (87.0%)		261 (87.3%)	79 (80.6%)		220 (86.3%)	84 (88.4%)	
Lobular	5 (9.1%)	7 (8.1%)	4 (2.2%)	0 (0.0%)		9 (3.0%)	7 (7.1%)		11 (4.3%)	0 (0.0%)	
Other	7 (12.7%)	11 (12.6%)	13 (7.3%)	10 (13.0%)		29 (9.7%)	12 (12.3%)		24 (9.4%)	11 (11.6%)	
Adjuvant chemotherapy	*(missing: 2)*				<0.001	*(missing: 2)*		0.237	*(missing: 1)*		0.007
No	22 (40.7%)	29 (33.7%)	36 (20.2%)	7 (9.1%)		75 (25.3%)	19 (19.4%)		63 (24.8%)	11 (11.6%)	
Yes	32 (59.3%)	57 (66.3%)	142 (79.8%)	70 (90.9%)		222 (74.7%)	79 (80.6%)		191 (75.2%)	84 (88.4%)	
Basal-like phenotype	*(missing:7)*				<0.001 <0.001	*(missing:7)*		0.924	*(missing:7)*		0.305
Basal-like	21 (38.2%)	48 (55.2%)	111 (64.5%)	54 (71.1%)		176 (59.9%)	58 (60.4%)		154 (61.4%)	62 (67.4%)	
Non-basal-like	34 (61.8%)	39 (44.8%)	61 (35.5%)	22 (28.9%)		118 (40.1%)	38 (39.6%)		97 (38.6%)	30 (32.6%)	
Molecular apocrine phenotype	*(missing: 28)*				<0.001 <0.001	*(missing: 28)*		0.024	*(missing: 24)*		0.051
Molecular apocrine	24 (50.0%)	38 (45.2%)	59 (35.1%)	12 (17.4%)		92 (32.9%)	41 (46.1%)		88 (36.8%)	22 (25.3%)	
Non-molecular apocrine	24 (50.0%)	46 (54.8%)	109 (64.9%)	57 (82.6%)		188 (67.1%)	48 (53.9%)		151 (63.2%)	65 (74.7%)	

Basal-like phenotype was defined by the expression of cytokeratin 5/6 and/or EGFR in >10% of tumor cells; molecular apocrine phenotype was considered in the case of positive staining for both androgen receptor and Forkhead box protein A1 (FoxA1) biomarkers in ≥1% of tumor cells.

### 3.4 TLS, immune cell populations and immune checkpoint components (univariate analysis)

TIL levels increased with PT-TLS abundance (p<0.001) and the presence of IT-TLS (p=0.002) and m-TLS (p<0.001) (**Table 3**). As the amount of PT-TLS increased, the proportion of cold tumors decreased and the proportion of excluded and hot tumors increased progressively (p<0.001). In the same way, the presence of IT-TLS and of TLS with CGC was significantly associated with hot inflammatory pattern (p=0.014 and p<0.001, respectively). Lastly, IT-TLS frequency was similar in the different PT-TLS abundance groups (p=0.121) and not related to the presence of CGC (p=0.239).

**Table 3:** Results of the univariate analysis of association between TLS and immune features.

	Peritumoral TLS					Intratumoral TLS			TLS		
	None	Little	Moderate	Abundant	p-value	No	Yes	p-value	Without CGC	With CGC	p-value
	n=55	n=87	n=178	n=77		n =299	n =98		n =255	n =95	
TILs					<0.001			0.002			<0.001
<30%	49 (89.1%)	68 (78.2%)	111 (62.4%)	33 (42.9%)		209 (69.9%)	52 (53.1%)		178 (69.8%)	40 (42.1%)	
≥30%	6 (10.9%)	19 (21.8%)	67 (37.6%)	44 (57.1%)		90 (30.1%)	46 (46.9%)		77 (30.2%)	55 (57.9%)	
Inflammatory pattern					<0.001			0.014			<0.001
Cold	48 (87.3%)	63 (72.4%)	93 (52.2%)	28 (36.4%)		187 (62.5%)	45 (45.9%)		158 (61.9%)	32 (33.7%)	
Excluded	4 (7.3%)	7 (8.1%)	27 (15.2%)	21 (27.2%)		41 (13.7%)	18 (18.4%)		29 (11.4%)	26 (27.4%)	
Hot	3 (5.4%)	17 (19.5%)	58 (32.6%)	28 (36.4%)		71 (23.8%)	35 (35.7%)		68 (26.7%)	37 (38.9%)	
Peritumoral TLS					–			0.121			<0.001
None	–	–	–	–		47 (15.7%)	8 (8.2%)		6 (2.4%)	2 (2.1%)	
Little	–	–	–	–		64 (21.4%)	23 (23.5%)		82 (32.2%)	5 (5.3%)	
Moderate	–	–	–	–		136 (45.5%)	42 (42.8%)		134 (52.5%)	44 (46.3%)	
Abundant	–	–	–	–		52 (17.4%)	25 (25.5%)		33 (12.9%)	44 (46.3%)	
m-TLS (with CGC)					<0.001			0.239			–
Absent	6 (75.0%)	82 (94.3%)	134 (75.3%)	33 (42.9%)		188 (74.6%)	67 (68.4%)		–	–	
Present	2 (25.0%)	5 (5.7%)	44 (24.7%)	44 (57.1%)		64 (25.4%)	31 (31.6%)		–	–	

TILs, tumor-inflitrating lymphocytes; TLS, tertiary lymphoid structures; m-TLS, mature-TLS; CGC, clear germinal center.

**Figure 3:**
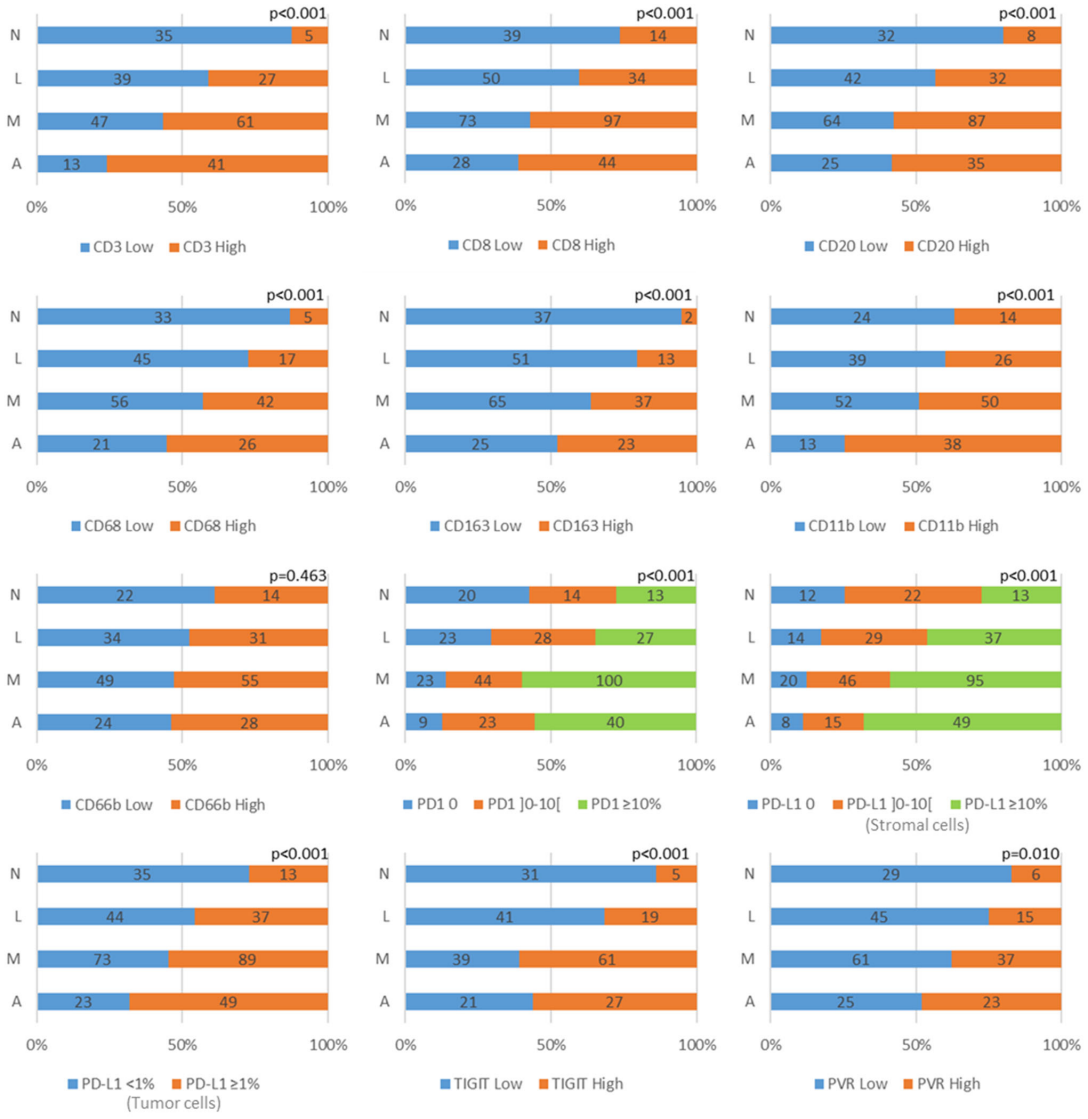
Graphical representation of the association between PT-TLS abundance (N: none, L: little, M: moderate, A: abundant) and the expression of various lymphoid (CD3, CD8, CD20), myeloid (CD68, CD163, CD11b, CD66b) and immune checkpoint (PD1, PD-L1 in stromal and tumor cells, TIGIT and PVR) markers. Low and high density categories were defined according to the median value of each biomarker, and for PVR, by grouping the two first terciles versus the third.

To decipher the interplay between TLS and other immune cell components, an univariate analysis of TLS features (PT-TLS abundance, IT-TLS presence and CGC presence as a surrogate of m-TLS) and various immune cell populations in the tumor parenchyma was carried out (**Figure 3**; **Supplementary Table S1**). The results showed that with the exception of the CD66+ cell population, tumors with higher density of the different immune cell types also presented higher PT-TLS abundance. Specifically, PT-TLS abundance and IT-TLS and m-TLS presence were higher in tumors with high CD3+ or CD8+ cell density. Moreover, PT-TLS abundance and IT-TLS presence increased in tumors with high B-cell infiltrate (CD20+ cells; p<0.001 for both). Similarly, PT-TLS abundance and m-TLS presence, but not IT-TLS presence, increased in tumors with high density of CD68+ and CD163+ macrophages and CD11b+ cell infiltrates. Remarkably, the percentage of tumors with high infiltration of T, B cells and macrophages increased with PT-TLS abundance (**Figure 3**).

Then, TLS abundance/features and the expression of the immune checkpoint components PD1/PD-L1 and TIGIT/PVR was investigated by univariate analysis (**Figure 3**; **Supplementary Table S1**). PT-TLS abundance was higher in TNBC samples with higher expression of PD1 (p<0.001) and PD-L1 (p<0.001) by stromal cells, probably because they are mainly expressed by lymphoid and myeloid cells that also were associated with PT-TLS presence. Similarly, PT-TLS abundance (p<0.001) and m-TLS presence (p<0.001), but not IT-TLS presence (p=0.401), were increased in samples with high PD-L1 expression by tumor cells. Lastly, PT-TLS abundance (p<0.001), IT-TLS (p=0.026) and m-TLS (p=0.002) presence were higher in samples with high TIGIT expression, but only PT-TLS abundance was associated with high PVR expression (p=0.010).

### 3.5 Survival analysis

In univariate analysis (**Table 4**), the classical prognostic factors (tumor size, lymph node status, pathological stage and adjuvant chemotherapy) were significantly associated with RFS. High TIL levels and hot tumors were associated with longer RFS (HR=0.474, p=0.004 and HR=0.574, p=0.036, respectively). Moreover, RFS was longer in patients with tumors showing moderate (HR: 0.444, p=0.002) or abundant PT-TLS (HR: 0.177, p<0.001), but was similar in patients with/without IT-TLS and m-TLS. High density of CD3+ (HR: 0.555, p=0.018), CD20+ (HR: 0.482, p=0.002) and CD163+ (HR: 0.514, p=0.039) cell infiltrates was associated with better RFS as well as high levels of PD-L1 and PVR in tumor cells (HR: 0.626, p=0.034 and HR: 0.538, p=0.034, respectively). RFS tended to be better in patients with TNBC showing high density of CD11b+ and TIGIT+ cells (HR: 0.611, p=0.056 and HR: 0.627, p=0.067, respectively).

**Table 4:** Results of the univariate analysis for relapse-free survival.

	Relapse-free survival		
	HR	p-value	95% CI
Age (Years)			
<59.2	1		
≥59.2	1.270	0.253	0.843 - 1.914
Tumor size			
T1	1		
T2	2.492	<0.001	1.558 - 3.984
T3/T4	6.316	<0.001	3.218 - 12.396
Node status			
N-	1		
N+	3.619	<0.001	2.39 - 5.479
Pathological stage			
I	1		
II	2.334	0.005	1.298 – 4.195
III	9.522	<0.001	5.213 – 17.393
Histological grade			
I / II	1		
III	0.898	0.665	0.553 - 1.459
HER2			
Null	1		
Low	1.113	0.662	0.689 - 1.796
Histology			
NST	1		
Lobular	1.338	0.500	0.573 - 3.123
Other	0.659	0.279	0.310 - 1.401
Adjuvant chemotherapy			
No	1		
Yes	0.369	<0.001	0.244 - 0.558
Basal-like phenotype			
Non-basal-like	1		
Basal-like	0.752	0.179	0.495 - 1.14
Molecular apocrine phenotype			
Non-molecular apocrine	1		
Molecular apocrine	1.352	0.157	0.89 - 2.055
TILs			
<30%	1		
≥30%	0.474	0.004	0.286 - 0.786
Inflammatory pattern			
Cold	1		
Excluded	0.595	0.129	0.305 - 1.162
Hot	0.574	0.036	0.342 - 0.965
Peritumoral TLS			
None	1		
Little	0.584	0.068	0.328 - 1.041
Moderate	0.444	0.002	0.263 - 0.751
Abundant	0.177	<0.001	0.076 - 0.416
m-TLS (with CGC)			
Absence	1		
Presence	0.650	0.133	0.371 - 1.14
Intratumoral TLS			
Absence	1		
Presence	1.051	0.837	0.653 - 1.693
CD3+ cells*			
Low	1		
High	0.555	0.018	0.341 - 0.903
CD8+ cells*			
Low	1		
High	0.904	0.63	0.598 - 1.365
CD20+ cells*			
Low	1		
High	0.482	0.002	0.303 - 0.769
CD68+ cells*			
Low	1		
High	0.717	0.23	0.417 - 1.233
CD163+ cells*			
Low	1		
High	0.514	0.039	0.273 - 0.967
CD11b+ cells*			
Low	1		
High	0.611	0.056	0.369 - 1.012
CD66b+ cells*			
Low	1		
High	1.082	0.75	0.667 - 1.755
PD1+ cells			
0	1		
]0-10[	0.884	0.676	0.496 - 1.574
≥10%	0.845	0.544	0.490 - 1.457
PD-L1+ tumor cells			
<1%	1		
≥1%	0.626	0.034	0.406 - 0.965
PD-L1+ stromal cells			
0	1		
]0-10[	1.395	0.302	0.742 - 2.623
≥10%	0.682	0.241	0.360 - 1.292
TIGIT+ cells*			
Low	1		
High	0.627	0.067	0.381 - 1.033
PVR+ cells^§^			
Low	1		
High	0.538	0.034	0.304 - 0.955

HR: hazard ratio; Basal-like phenotype was defined by the expression of cytokeratin 5/6 and/or EGFR in >10% of tumor cells; molecular apocrine phenotype was considered in the case of positive staining for both androgen receptor and Forkhead box protein A1 (FoxA1) biomarkers in ≥1% of tumor cells. TILs, tumor-inflitrating lymphocytes; TLS, tertiary lymphoid structures; m-TLS, mature-TLS; CGC, clear germinal center. Low and high density categories were defined according to the median* and for PVR, by grouping the two first terciles versus the third^§^ (see Materials and Methods).

**Figure 4:**
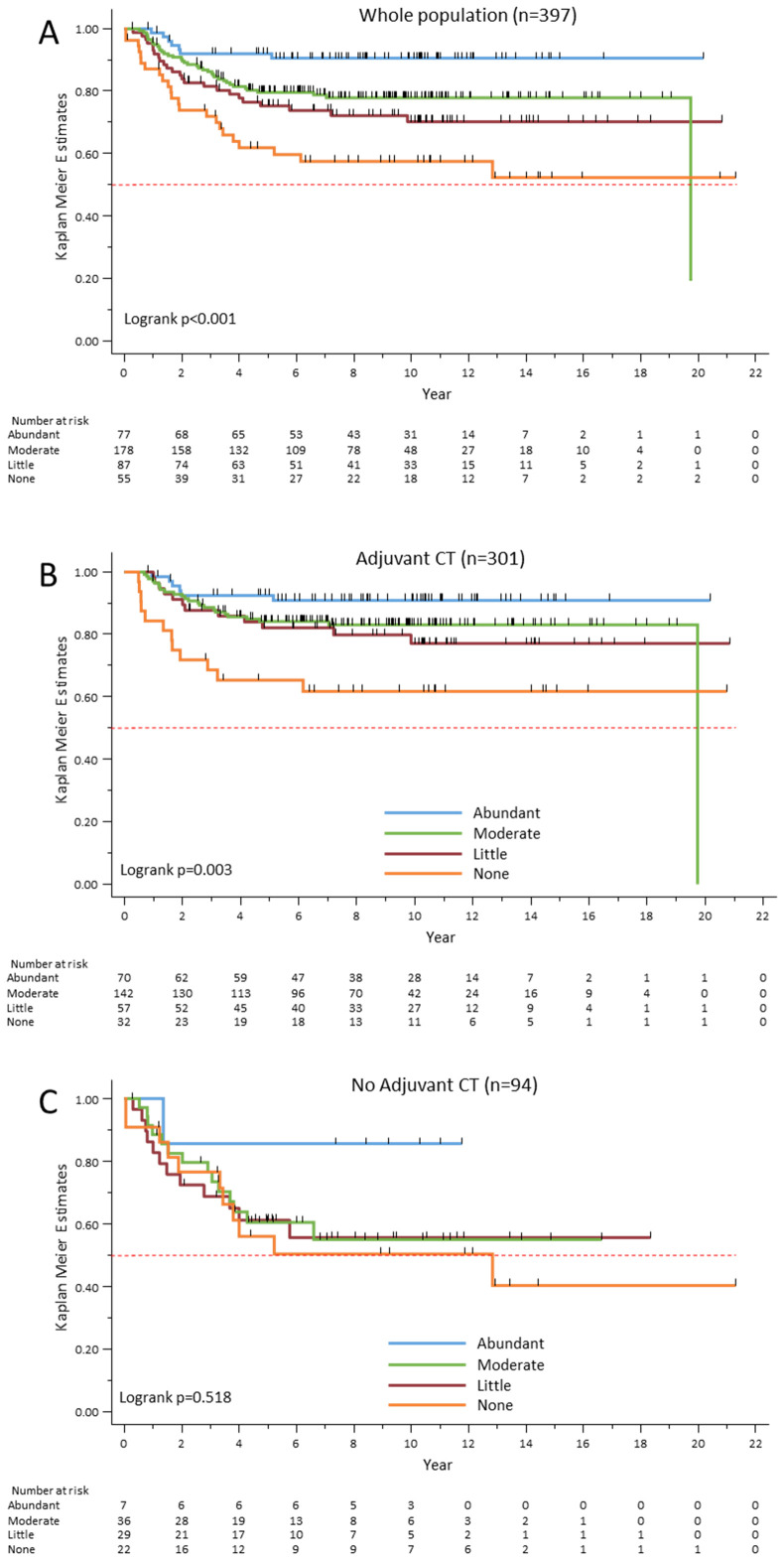
Relapse-free survival rates according to PT-TLS abundance in the whole cohort **(A)**, and in patients treated **(B)** or not **(C)** with adjuvant chemotherapy (CT).

The Kaplan–Meier survival analysis (**Figure 4A**) confirmed that RFS progressively increased with PT-TLS abundance. The correlation between TLS abundance and RFS was found for all phenotypic groups analyzed, with the exception of the apocrine molecular group, for which only patients with abundant PT-TLS showed excellent RFS (data not shown). Then, to determine the predictive impact of PT-TLS, RFS was compared in patients who received (n=301) or not (n=94) adjuvant chemotherapy. The Kaplan–Meier survival analysis showed that in the treated subgroup (**Figure 4B**), the risk of relapse progressively decreased with PT-TLS abundance (p=0.003). In the untreated subgroup, only patients with abundant PT-TLS showed a favorable outcome compared with patients with moderate, little or none PT-TLS (**Figure 4C**). However, due to the small number of patients, and particularly those with abundant PT-TLS, the difference was not statistically significant (p=0.518), even when patients with moderate, low or no PT-TLS were grouped together (p=0.151, data not shown). Thus, these results should be treated with caution. In summary, PT-TLS had a strong predictive value, with a clear gradient between PT-TLS abundance and favorable clinical outcome, whereas their prognostic value seems to be restricted to the highest TLS amount.

Multivariate analysis showed that besides the known prognostic factors of pathological stage and adjuvant chemotherapy, the abundance of PT-TLS was significantly correlated with better RFS (**Table 5**), whereas the classical TIL variable was not retained in the model.

**Table 5:** Results of the multivariate analysis for relapse-free survival.

	Relapse-free survival (n=395)		
	HR	p-value	95% CI
Peritumoral TLS			
None	1		
Little	0.617	0.110	0.341 - 1.116
Moderate	0.663	0.161	0.373 - 1.178
Abundant	0.310	0.012	0.124 - 0.772
Pathological stage			
I	1		
II	2.361	0.004	1.312 - 4.251
III	8.776	<0.001	4.824 - 15.966
Adjuvant chemotherapy			
No	1		
Yes	0.434	<0.001	0.279 - 0.676

HR, hazard ratio; TLS, tertiary lymphoid structures.

We performed a separate statistical analysis, excluding the 42 cases that had hormone receptors between 1% and 9%. Results were similar in both populations (including or not the samples with ER and/or PR in the 1-9% range). In particular, the multivariate analysis shown that PT-TLS continue to be significantly associated with better RFS independently to other variables (data not shown).

## 4 Discussion

The goal of this study was to screen for the occurrence of PT-, IT- and m-TLS in a well-characterized cohort of 397 localized TNBC samples and determine their association with clinicopathological features, the tumor immune microenvironment and clinical outcome. Our results, obtained using HES-stained tumor sections, confirmed that TLS abundance, mostly at the tumor periphery, is a major independent prognostic maker besides other well-described factors, such as tumor size, lymph node involvement and adjuvant chemotherapy. Moreover, in our study, TLS outperformed TILs as a prognostic factor, although they are considered a major prognostic factor in early-stage TNBC (20).

The lack of standardized criteria to assess TLS most likely contributes to their infrequent analysis in routine clinical practice. Indeed, various morphological, phenotypic and localization features have been used to define TLS across studies. Histologically, TLS are defined as aggregates of >50 lymphoid cells, most often located at the invasive margin and more rarely within the tumor mass. Using this definition, TLS can be assessed during the routine histological examination. Phenotypically, TLS are characterized by central B-cell clusters surrounded by T cells. In this case, IHC is required to define TLS. Lastly, TLS are structurally separated into two classes: i) m-TLS that are organized as secondary follicles, with CGC that include follicular DCs and high endothelial venules and ii) immature TLS that are poorly shaped, without CGC and DCs. The evaluation of TLS maturation requires multiparametric analyses and this is challenging in routine practice. Functionally, m-TLS are involved in clonal B-cell amplification, differentiation and affinity maturation through somatic hypermutation and isotype switching, leading to the generation of memory B cells that sustain the long-term immune responses (10, 28) and plasma cells that secrete locally large amounts of high-affinity antibodies.

In this study, we assessed TLS using HES-stained TNBC sections and the method described by Lee et al. (14), i.e. by quantifying the tumor front occupied by TLS. We think that this method is easier to implement and importantly, more reproducible than counting TLS. Indeed, TLS do not always appear as dense, nodular lymphoid aggregates, but also as more or less diffuse sheets. Using this approach, we could detect TLS in 88.1% of the included TNBC samples (350/397), mainly at the tumor front (342/350). In 90 samples, TLS were also present within the tumor. In the remaining 8 samples, TLS were exclusively localized within the tumor parenchyma. Our results are in accordance with those by Vanhersecke et al. (18) and Lee et al. (14) who observed TLS predominantly at the tumor front in various cancer types, including TNBC. Interestingly, PT-TLS, but not IT-TLS, were associated with various clinicopathological data. Conversely, CGC presence did not bring any additional value compared with PT-TLS detection. In our study, CGC presence was used as a surrogate marker of m-TLS. More sophisticated techniques, such as multiplexed immunofluorescence, showed that CGC are specific of m-TLS. Conversely, the analysis of HES-stained sections is not sensitive enough (18). TLS without detectable GCC on HES-stained sections may in fact be classified as m-TLS when specific biomarkers (e.g. the DC marker CD23) are used (18). We hypothesize that the probability of having an m-TLS in the whole tumor (and not just in the tumor section studied) increases with TLS abundance. In agreement, in our series, 46.3% of samples in which TLS with CGC were detected had abundant PT-TLS compared with 12.9% of samples without CGC. This could explain why our results show that PT-TLS assessment (regardless of their degree of maturity) using simple routine HES-stained tumor sections can provide prognostic information in addition to the classic clinicopathological factors taken into account for clinical management.

The strength of our study lies in the fact that in a large cohort of early TNBC samples, we evaluated TLS using a readily method accessible in routine pathology in parallel of different immune biomarkers, including immune checkpoints, immune patterns (hot, cold, excluded), TILs and various clinicopathological features. Like Yazaki et al. (17), we found that PT-TLS abundance was higher in TNBC samples with higher densities of T and B cells and also of myeloid cell populations (CD68+, CD163+ and CD11b+ cells). Although surprising, the positive association between PT-TLS and CD163+ cells, commonly related to M2 macrophage polarization (29), had already been reported in cervical tumors by Gorvel et al. (30). They showed myeloid cell activation, particularly higher CD163+ density in TLS-positive tumors compared with TLS-negative samples. The biological impact is unclear.

We also found that PT-TLS abundance increased with the expression of the immune checkpoint factors PD1/PD-L1 and TIGIT/PVR. PD1 and TIGIT are expressed by activated T cells, reflecting the activation of T cells that may be consistent with TLS presence. Several mechanisms are responsible for the expression or upregulation of PD-L1 on tumor cells. One of these is the release of IFN-γ by activated T cells that upon binding to IFNR, triggers the activation of the interferon regulatory factor axis. Thus, IFN-γ induces PD-L1 expression through a negative feedback loop that in turn suppresses TIL activities (31). PVR is very weakly expressed in normal cells and tissues, but can be upregulated in some conditions, such as cellular stress, inflammatory cytokine stimulation and cell proliferation [reviewed in (32, 33)]. This could explain why PT-TLS abundance increased with PVR expression.

Altogether, our results emphasize the intricate balance between the tumor and the host immune system in which TLS act as weapon factories at the tumor site. Indeed, we observed a strong correlation between PT-TLS abundance and favorable outcome. As the multivariate analysis retained only PT-TLS among all other immune cell populations included in the model for the RFS analysis, particularly TILs, we hypothesize that their presence might give a more global picture of the tumor/host balance than TILs alone. In our series, PT-TLS had a strong predictive value, whereas their value seemed to be limited to tumors with abundant PT-TLS. The latter result should be treated with caution, given the small number of samples from patients who did not receive adjuvant chemotherapy (n=94). In a series of 125 patients with early stage TNBC who did not receive adjuvant chemotherapy, Yazaki et al. failed to demonstrate any prognostic impact of PT-TLS (17). However, they defined the clinical outcome as invasive disease-free survival (i.e. the time from surgery to the first relapse, contralateral breast cancer or death from any cause). Conversely, we defined RFS as the time from the date of surgery to the date of the first local and/or distant tumor relapse because we considered that in this population with a long follow-up, a significant number of deaths was not linked to the cancer.

After the recent approval of immunotherapy for patients with early stage or metastatic TNBC, TLS could become a valuable biomarker of the response. Vanhersecke et al. showed that the presence of m-TLS correlates with the response to immune checkpoint inhibitors (ICI) in various solid tumor types (11). However, in their study, only 6 of the 328 patients included had breast cancer. Therefore, m-TLS predictive value of ICI benefit in TNBC needs to be confirmed because currently, there is no validated predictive biomarker to identify the patients sensitive to ICI in the context of neo-adjuvant treatment. Indeed, the results of the KEYNOTE-522 study showed that patients with early TNBC benefit from the addition of pembrolizumab to chemotherapy, regardless of their PD-L1 score (8). However, in this setting, TLS will have to be identified on biopsies, which represents a major challenge, as the probability of detecting m-TLS in biopsies is 6.1 times lower than in surgical specimens (18). Artificial intelligence applied to histopathology (34) and radiomic analysis (35) could help to identify these histological structures before ICI use in the neo-adjuvant setting.

Our results suggest that beside TIL quantification, the evaluation of PT-TLS on simple HES-stained sections could improve the clinical management of patients with early stage TNBC. In particular, the absence of TLS at the periphery of the tumor indicates a poor prognosis in patients receiving adjuvant chemotherapy. This subgroup of patients, identified on this variable, could thus be considered for adjuvant treatment escalation, representing an unmet medical need. The clinical value of PT-TLS as a predictor of the response to neoadjuvant treatments, including chemoimmunotherapies needs to be further evaluated.

## Data Availability

The raw data supporting the conclusions of this article will be made available by the authors, without undue reservation.
