# Supplementary figures and images for: Prognostic value of tertiary lymphoid structures in triple-negative breast cancer: integrated analysis with the tumor microenvironment and clinicopathological features
Source: Front Immunol. 2024 Dec 12;15:1507371. doi: 10.3389/fimmu.2024.1507371 (PMC11669358; doi:10.3389/fimmu.2024.1507371)

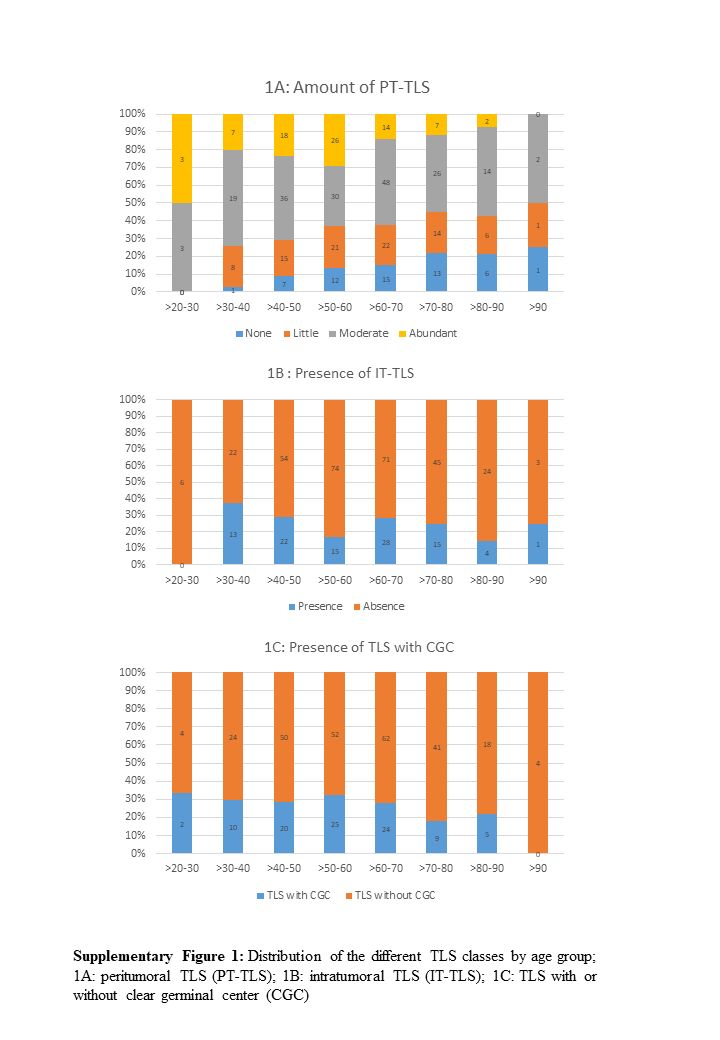

Supplement: Supplementary file 1 [file Image1.tif]

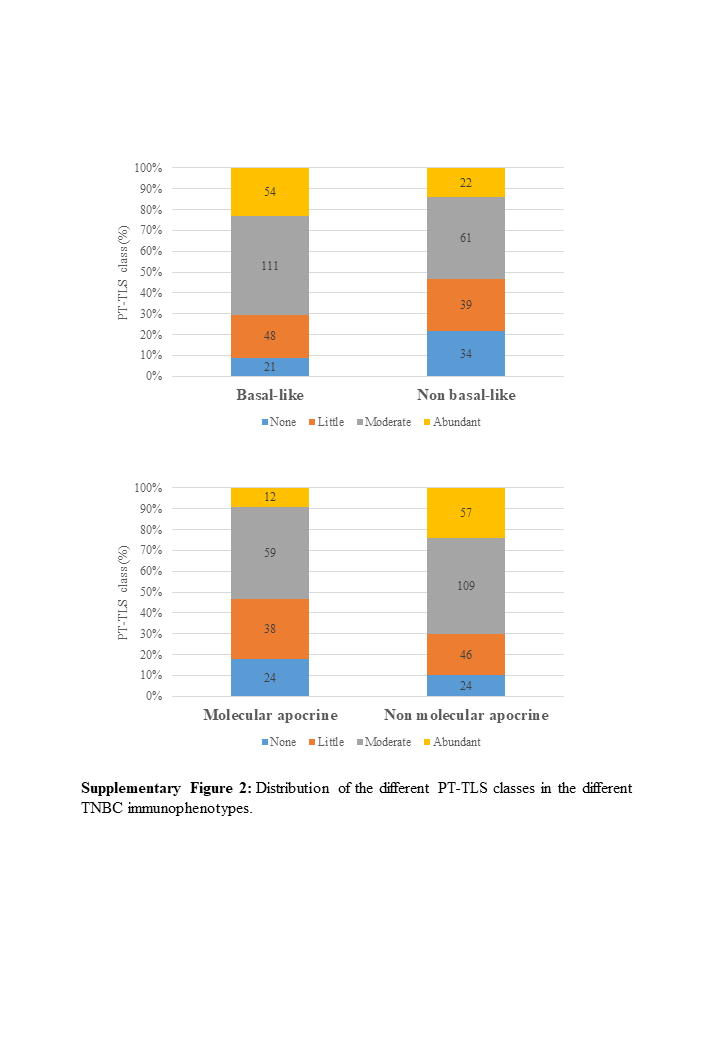

Supplement: Supplementary file 2 [file Image2.tif]
