# Supplementary material for: Prognostic value of tertiary lymphoid structures in triple-negative breast cancer: integrated analysis with the tumor microenvironment and clinicopathological features
Source: Front Immunol. 2024 Dec 12;15:1507371. doi: 10.3389/fimmu.2024.1507371 (PMC11669358; doi:10.3389/fimmu.2024.1507371)
Supplement: Supplementary file 3 [file Table1.docx]

| **Suppl Table 1: Results of the univariate analysis between TLS and various immune cell populations** | | | | | | | | | | | | | | |
| --- | --- | --- | --- | --- | --- | --- | --- | --- | --- | --- | --- | --- | --- | --- |
|  | **Peri-tumoral TLS** | | | |  |  | **Intra-tumoral TLS** | |  |  | **TLS** | | |  |
|  | **None** | **Little** | **Moderate** | **Abundant** | **p-value** |  | **No** | **Yes** | **p-value** |  | **Without CGC** | **With CGC** | | **p-value** |
|  | **n=55** | **n=87** | **n=178** | **n=77** |  |  | **n =299** | **n =98** |  |  | **n =255** | **n =95** | |  |
| **CD3+ cells*** | *(missing: 129)* |  |  |  | <0.001 |  | *(missing: 129)* |  | 0.036 |  | *(missing: 116)* |  | | 0.011 |
| Low | 35 (87.5%) | 39 (59.1%) | 47 (43.5%) | 13 (24.1%) |  |  | 107 (53.8%) | 27 (39.1%) |  |  | 83 (49.1%) | 20 (30.8%) | |  |
| High | 5 (12.5%) | 27 (40.9%) | 61 (56.5%) | 41 (75.9%) |  |  | 92 (46.2%) | 42 (60.9%) |  |  | 86 (50.9%) | 45 (69.2%) | |  |
|  |  |  |  |  |  |  |  |  |  |  |  |  | |  |
| **CD8+ cells*** | *(missing: 18)* |  |  |  | <0.001 |  | *(missing: 18)* |  | 0.012 |  | *(missing: 16)* |  | | 0.003 |
| Low | 39 (73.6%) | 50 (59.5%) | 73 (42.9%) | 28 (38.9%) |  |  | 153 (53.9%) | 37 (38.9%) |  |  | 126 (52.1%) | 31 (33.7%) | |  |
| High | 14 (26.4%) | 34 (40.5%) | 97 (57.1%) | 44 (61.1%) |  |  | 131 (46.1%) | 58 (61.1%) |  |  | 116 (47.9%) | 61 (66.3%) | |  |
|  |  |  |  |  |  |  |  |  |  |  |  |  | |  |
| **CD20+ cells*** | *(missing: 72)* |  |  |  | <0.001 |  | *(missing: 72)* |  | <0.001 |  | *(missing: 58)* |  | | 0.133 |
| Low | 32 (80.0%) | 42 (56.8%) | 64 (42.4%) | 25 (41.7%) |  |  | 135 (56.2%) | 28 (32.9%) |  |  | 103 (48.6%) | 31 (38.8%) | |  |
| High | 8 (20.0%) | 32 (43.2%) | 87 (57.6%) | 35 (58.3%) |  |  | 105 (43.8%) | 57 (67.1%) |  |  | 109 (51.4%) | 49 (61.2%) | |  |
|  |  |  |  |  |  |  |  |  |  |  |  |  | |  |
| **CD68+ cells*** | *(missing: 152)* |  |  |  | <0.001 |  | *(missing: 152)* |  | 0.653 |  | *(missing: 137 )* |  | | 0.001 |
| Low | 33 (86.8%) | 45 (72.6%) | 56 (57.1%) | 21 (44.7%) |  |  | 116 (64.1%) | 39 (60.9%) |  |  | 104 (66.2%) | 23 (41.1%) | |  |
| High | 5 (13.2%) | 17 (27.4%) | 42 (42.9%) | 26 (55.3%) |  |  | 65 (35.9%) | 25 (39.1%) |  |  | 53 (33.8%) | 33 (58.9%) | |  |
|  |  |  |  |  |  |  |  |  |  |  |  |  | |  |
| **CD163+ cells*** | *(missing: 144)* |  |  |  | <0.001 |  | *(missing: 144)* |  | 0.240 |  | *(missing: 130)* |  | | 0.005 |
| Low | 37 (94.9%) | 51 (79.7%) | 65 (63.7%) | 25 (52.1%) |  |  | 136 (72.3%) | 42 (64.6%) |  |  | 115 (71.9%) | 31 (51.7%) | |  |
| High | 2 (5.1%) | 13 (20.3%) | 37 (36.3%) | 23 (47.9%) |  |  | 52 (27.7%) | 23 (35.4%) |  |  | 45 (28.1%) | 29 (48.3%) | |  |
|  |  |  |  |  |  |  |  |  |  |  |  |  | |  |
| **CD11b+ cells*** | *(missing: 141)* |  |  |  | <0.001 |  | *(missing: 141)* |  | 0.114 |  | *(missing: 127 )* |  | | 0.019 |
| Low | 24 (63.2%) | 39 (60.0%) | 52 (51.0%) | 13 (25.5%) |  |  | 90 (47.1%) | 38 (58.5%) |  |  | 87 (53.7%) | 22 (36.1%) | |  |
| High | 14 (36.8%) | 26 (40.0%) | 50 (49.0%) | 38 (74.5%) |  |  | 101 (52.9%) | 27 (41.5%) |  |  | 75 (46.3%) | 39 (63.9%) | |  |
|  |  |  |  |  |  |  |  |  |  |  |  |  | |  |
| **CD66b+ cells*** | *(missing: 140)* |  |  |  | 0.463 |  | *(missing: 140)* |  | 0.451 |  | *(missing: 124)* |  | | 0.665 |
| Low | 22 (61.1%) | 34 (52.3%) | 49 (47.1%) | 24 (46.2%) |  |  | 99 (51.6%) | 30 (46.2%) |  |  | 82 (50.0%) | 29 (46.8%) | |  |
| High | 14 (38.9%) | 31 (47.7%) | 55 (52.9%) | 28 (53.8%) |  |  | 93 (48.4%) | 35 (53.8%) |  |  | 82 (50.0%) | 33 (53.2%) | |  |
|  |  |  |  |  |  |  |  |  |  |  |  |  | |  |
| **PD1+ cells** | *(missing: 33)* |  |  |  | <0.001 |  | *(missing: 33)* |  | 0.115 |  | *(missing: 26)* |  | | 0.010 |
| 0 | 20 (42.5%) | 23 (29.5%) | 23 (13.8%) | 9 (12.5%) |  |  | 63 (23.0%) | 12 (13.3%) |  |  | 50 (21.7%) | 8 (8.6%) | |  |
| ]0-10[ | 14 (29.8%) | 28 (35.9%) | 44 (26.3%) | 23 (31.9%) |  |  | 82 (29.9%) | 27 (30.0%) |  |  | 71 (30.7%) | 27 (29.0%) | |  |
| ≥10% | 13 (27.7%) | 27 (34.6%) | 100 (59.9%) | 40 (55.6%) |  |  | 129 (47.1%) | 51 (56.7%) |  |  | 110 (47.6%) | 58 (62.4%) | |  |
|  |  |  |  |  |  |  |  |  |  |  |  |  | |  |
| **PD-L1+ tumor cells** | *(missing: 34)* |  |  |  | <0.001 |  | *(missing: 34)* |  | 0.401 |  | *(missing: 29)* |  | | <0.001 |
| <1% | 35 (72.9%) | 44 (54.3%) | 73 (45.1%) | 23 (31.9%) |  |  | 136 (49.5%) | 39 (44.3%) |  |  | 119 (51.3%) | 27 (30.3%) | |  |
| ≥1% | 13 (27.1%) | 37 (45.7%) | 89 (54.9%) | 49 (68.1%) |  |  | 139 (50.5%) | 49 (55.7%) |  |  | 113 (48.7%) | 62 (69.7%) | |  |
|  |  |  |  |  |  |  |  |  |  |  |  |  | |  |
| **PD-L1+ stromal cells** | *(missing: 37)* |  |  |  | <0.001 |  | *(missing: 37)* |  | 0.299 |  | *(missing: 32)* |  | | 0.134 |
| 0 | 12 (25.5%) | 14 (17.5%) | 20 (12.4%) | 8 (11.1%) |  |  | 42 (15.4%) | 12 (13.8%) |  |  | 33 (14.4%) | 11 (12.5%) | |  |
| ]0-10[ | 22 (46.8%) | 29 (36.2%) | 46 (28.6%) | 15 (20.8%) |  |  | 90 (33.0%) | 22 (25.3%) |  |  | 73 (31.7%) | 19 (21.6%) | |  |
| ≥10% | 13 (27.7%) | 37 (46.3%) | 95 (59.0%) | 49 (68.1%) |  |  | 141 (51.6%) | 53 (60.9%) |  |  | 124 (53.9%) | 58 (65.9%) | |  |
|  |  |  |  |  |  |  |  |  |  |  |  |  | |  |
| **TIGIT+ cells*** | *(missing: 153)* |  |  |  | <0.001 |  | *(missing: 153)* |  | 0.026 |  | *(missing: 136)* |  | | 0.002 |
| Low | 31 (86.1%) | 41 (68.3%) | 39 (39.0%) | 21 (43.7%) |  |  | 104 (58.4%) | 28 (42.4%) |  |  | 86 (55.8%) | 19 (31.7%) | |  |
| High | 5 (13.9%) | 19 (31.7%) | 61 (61.0%) | 27 (56.3%) |  |  | 74 (41.6%) | 38 (57.6%) |  |  | 68 (44.2%) | 41 (68.3%) | |  |
|  |  |  |  |  |  |  |  |  |  |  |  |  | |  |
| **PVR+ cells^§^** | *(missing: 156)* |  |  |  | 0.010 |  | *(missing: 156)* |  | 0.508 |  | *(missing: 138)* |  | | 0.081 |
| Low | 29 (82.9%) | 45 (75.0%) | 61 (62.2%) | 25 (52.1%) |  |  | 119 (67.6%) | 41 (63.1%) |  |  | 103 (67.8%) | 33 (55.0%) | |  |
| High | 6 (17.1%) | 15 (25.0%) | 37 (37.8%) | 23 (47.9%) |  |  | 57 (32.4%) | 24 (36.9%) |  |  | 49 (32.2%) | 27 (45.0%) | |  |
|  | | | | | | | | | | | | |  | |
| TLS: tertiary lymphoid structures; CGC: clear germinal center. Low and high density categories were defined according to the median* and for PVR, by grouping the two first terciles versus the third^§^ (see Materials and Methods) | | | | | | | | | | | | | | |
